# Vitamin A supplementation coverage and its associated factors among children 6–59 months of age in Ethiopia: a systematic review and meta-analysis

**DOI:** 10.3389/fpubh.2025.1496931

**Published:** 2025-04-08

**Authors:** Wubet Tazeb Wondie, Alemu Birara Zemariam, Gezahagn Demsu Gedefaw, Gebeyehu Lakew, Eyob Getachew, Berihun Agegn Mengistie, Adamu Ambachew Shibabaw, Alex Ayenew Chereka, Gemeda Wakgari Kitil, Amlaku Nigusie Yirsaw, Gebrehiwot Berie Mekonnen

**Affiliations:** ^1^Department of Pediatrics and Child Health Nursing, College of Health Sciences and Referral Hospital, Ambo University, Ambo, Ethiopia; ^2^Department of Pediatrics and Child Health Nursing, School of Nursing, College of Medicine and Health Science Woldia University, Woldia, Ethiopia; ^3^Department of Neonatal Health Nursing, School of Nursing, College of Medicine and Health Science, University of Gondar, Gondar, Ethiopia; ^4^Department of Health Promotion and Health Behavior, Institute of Public Health, College of Medicine and Health Sciences, University of Gondar, Gondar, Ethiopia; ^5^Department of General Midwifery, School of Midwifery, College of Medicine and Health Sciences, University of Gondar, Gondar, Ethiopia; ^6^Department of Health Informatics, College of Health Science, Mettu University, Mettu, Ethiopia; ^7^Department of Midwifery, College of Health Science, Mettu University, Mettu, Ethiopia; ^8^Department of Pediatrics and Child Health Nursing, College of Health Science, Debre Tabor University, Debre Tabor, Ethiopia

**Keywords:** associated factors, children 6–59 months’, coverage, Ethiopia, vitamin A supplementation

## Abstract

**Background:**

Vitamin A supplementation is a key strategy for preventing the consequences of vitamin A deficiency and childhood illnesses, notably in countries where vitamin A deficiency is a public health issue. However, studies in Ethiopia are inconsistent. Hence, this meta-analysis assessed coverage of vitamin A supplementation and associated factors among children aged 6–59 months in Ethiopia.

**Methods:**

A search of articles from databases (PubMed, Science Direct, African Index Medicus, and HINARI), and search engines (Google Scholar, Google, and Worldwide Science) was done. All observational studies that report vitamin A supplementation and/or associated factors among children were included. The Joana Brigs quality appraisal checklist was used. To estimate the pooled effect size a random effect model was used. Heterogeneity was evaluated using *I*^2^-test and Cochrane Q statistics. Subgroup and sensitivity analyses were conducted. Publication bias was assessed using Egger’s test and funnel plot.

**Results:**

A total of 14 studies, involving 43,047 children aged 6–59 months, were included. The pooled vitamin A supplementation coverage was 54.88% (95% CI: 47.34–62.42). The lowest coverage 43.71%% (95% CI: 42.71–45.14) was among children 6–35 months. Four or more antenatal care (AOR: 1.79, 95%CI: 1.59–2.01), Postnatal care (AOR: 1.43, 95% CI: 1.24–1.66), delivery at health facilities (AOR: 1.14 95%CI: 1.02–1.28), media exposure (AOR: 1.19, 95% CI: 1.08–1.31), time to reach health facilities (AOR: 1.90, 95% CI: 1.11–3.24), information about VAS (AOR: 2.99, 95%CI: 1.72–5.20), maternal secondary education and above (AOR: 1.32, 95% CI: 1.07–1.64), and (AOR: 2.31, 95% CI: 1.31–4.09) respectively, and fathers education above secondary school (AOR:1.92, 95% CI: 1.13–3.26) were significant factors.

**Conclusion:**

The pooled vitamin A supplementation coverage is significantly below the WHO’s recommendation of 80%. Antenatal care, postnatal care, health facilities delivery, media exposure, time to reach health facilities, Information about VAS, maternal and paternal secondary education, and above increase VAS. Hence, the national nutritional program is better to increase awareness of the community about VAS, particularly targeting parents with low educational status and no antenatal and postnatal care through social media and community meetings. Additionally, the EPI program should strengthen outreach supplementations including door-to-door distribution to address older children and socio-economically disadvantaged populations.

**Systematic review registration:**

identifier CRD42024576200.

## 1 Introduction

Vitamin A is an essential micronutrient required for normal vision, reproduction, healthy immune function, growth, and development (1, 2). However, inadequate intake of vitamin A increases the risk of mortality, and visual impairment, and weakens the body’s ability to fight off infection, notably in children (3). On the other hand, vitamin A supplementation (VAS) reduces all causes of mortality by 25% (4, 5), diarrhea-related mortality by 30%% (5), and measles and other respiratory infections (6).

In low and middle-income countries (LMIC), VAS coverage is below 70% (7), and vitamin A deficiency affects one-third of children, particularly in Sub-Saharan Africa (SSA) (8). The most severe outcomes of vitamin A deficiency such as corneal xerophthalmia, severe illness, and death are primarily found in children aged 6–59 months, making this population an urgent priority for vitamin A supplementation (VAS) (9). In areas where vitamin A deficiency is a significant public health concern, to reduce morbidity and mortality associated with vitamin A deficiency, the World Health Organization (WHO) recommends VAS for all children aged 6–59 months (10, 11). In such regions, an 80% coverage rate is recommended (9), because VAS is a cheap, safe, and cost-effective way to eliminate this nutritional deficiency and improve child survival (11).

However, VAS coverage remains below the recommended level, with notable geographical disparities. For instance, coverage rates in South Asia are 62%, in West and Central Africa 71%, in East and South Africa 57%, and in the East Asia and Pacific region 20% (12). In Sub-Saharan Africa, only 56.3% of children were received VAS (13). Coverage also varies widely across low and middle-income countries (LMIC), with countries like Mozambique 42.8%, Senegal 46.1%, Sierra Leone 86.9% (14), Guinea 68% (15), Mali 80% (16), Nigeria 43% (17). Tanzania 53.18% (18), Cambodia 42.8% (19), India 60.5% (20), and Brazil 41.5% (21). This indicates that the coverage is below the optimal level in many regions (22). These studies revealed that child age (6–12 months and 35–59 months) (23, 24), exposure to mass media (16, 18, 22, 25, 26), fathers educational status (16, 25, 27), mothers employment status (17, 18), antenatal care (17, 18), delivery in a health facility (17, 18), mothers secondary educational level (17, 19, 21, 25), receiving information from health care workers and peers (28), household wealth (17), place of residence (29), mothers with education secondary school are factors that affect vitamin A supplementation (23, 28, 29).

VAS has been a global health strategy, often integrated with routine immunization and campaigns to reduce the burden of vitamin A deficiency and its severe consequences (9). Various efforts have been implemented to enhance the coverage of vitamin A and mitigate the effect of vitamin A deficiency. For example, WHO recommends supplementation for every child every 4–6 months, and in SSA, door-to-door distribution has been used successfully to achieve high VAS coverage (30). In Ethiopia, the national nutritional program has promoted supplementation, and over the past two decades, the Ministry of Health has introduced different strategies to enhance VAS through health extension programs and other campaign-based approaches such as enhanced outreach strategy, community health days, and periodic supplementation (31, 32). The VAS program has now been integrated into the routine immunization program (32). Additionally, Ethiopia’s first and second transformation Plans aimed to increase VAS coverage in children to 95% by the end of 2020 (33, 34).

Despite these efforts, VAS coverage and vitamin A deficiency remain a significant public health challenge in Ethiopia (35, 36). In Ethiopia, several studies investigated VAS coverage and contributing factors at the district level and through national surveys. However, the findings on VAS coverage and the contributing factors are fragmented and inconsistent, ranging from 36.2% (37) to 83.1% (38). These figures do not provide comprehensive and robust conclusions for stakeholders. Therefore, the present meta-analysis assesses the vitamin A supplementation coverage and associated factors among children 6–59 months of age in Ethiopia. The findings of this study will help to prevent vitamin A deficiency and its related consequences by identifying factors that affect VAS and by providing evidence-based information to policymakers and program implementers. Furthermore, it will serve as a baseline data for future researchers.

## 2 Methods

### 2.1 Protocol registration and search strategy

The preferred reporting items for systematic review and meta-analysis (PRISMA 20) guideline was used for reporting the findings of this study (39) (Supplementary material 1). The protocol registration number of this study was CRD42024576200 which is available from https://www.crd.york.ac.uk/prospero/#recordDetails.

### 2.2 Searching strategy and study selection

A systematic search of studies was conducted by two authors to identify studies that report VAS coverage and/or associated factors among children 6–59 of age months in Ethiopia. The search of articles was carried out in databases such as PubMed, African Index Medicus (AIM), Science Direct, and HINARI using Medical Subject Heading (MeSH) terms and relevant keywords. Additionally, a manual search of articles was carried out using web search engines such as Google Scholar, Google, and Worldwide Science. The reference lists of included studies were reviewed, and gray literature from university repositories was examined. The Boolean operators AND, OR and Asterisks and the following terms and phrases was used during searching as follows: ((((((((((Coverage[Title/Abstract]) OR (“Vaccination Coverage”[Mesh])) OR (Prevalence[Title/Abstract])) OR (Magnitude[Title/Abstract])) OR (Proportion[Title/Abstract])) OR (Uptake[Title/Abstract])) OR (Differential[Title/Abstract])) OR (“Micro-nutrient intake”[Title/Abstract])) AND (((((((((“Associated factors”[Title/Abstract])) OR (“Precipitating Factors”[Mesh])) OR (Determinants[Title/Abstract])) OR (“Epidemiologic factors”[Title/Abstract])) OR (Predictors[Title/Abstract])) OR (“Risk factors”[Title/Abstract])) OR (Barriers[Title/Abstract])) OR (Facilitators[Title/Abstract]))) AND (((((((“Vitamin A supplementation”[Title/Abstract]) OR (“vitamin A”[MeSH Terms])) OR (Vitamin A[Title/Abstract])) OR (“Retinol supplementation”[Title/Abstract])) OR (“Pro vitamin A “[Title/Abstract])) OR (“Vitamin A Supplement*”[Title/Abstract])) OR (“Dietary supplementation”[Title/Abstract]))) AND ((((((((“Children 6–59 months”[Title/Abstract]) OR (“child”[MeSH Terms])) OR (Child*[Title/Abstract])) OR (Infants*[Title/Abstract])) OR (“Preschool children”[Title/Abstract])) OR (“Under five years’ children”[Title/Abstract])) OR (“Children 6–36 months”[Title/Abstract])) OR (“Children 6–24 months”[Title/Abstract])) AND (Ethiopia[Title/Abstract]). A search of articles from databases and search engines was done from August 3, 2024, to August 12, 2024. All eligible studies published up to August 12, 2024, were included (Supplementary material 2).

### 2.3 Eligibility criteria

This study includes all observational studies (cross-sectional, cohort, and case–control studies) written in the English language that report the vitamin A supplementation coverage among children 6–59 months of age in Ethiopia up to August 12, 2024. Studies that report only determinants of VAS were included to identify those specific determinants. The included studies followed the PECO framework: P = Population aged 6–59 months, E = children who receive vitamin A, C = children who do not receive vitamin A, O = vitamin A coverage and associated factor. On the other hand, Studies that do not report the outcome variables, abstract, and/or full text were excluded after two email attempts to contact the corresponding authors. Additionally, anonymous reports, editorials, and purely qualitative studies that do not report the proportion or determinants of VAS were also excluded.

### 2.4 The outcome of interest ascertainment

This study has two outcomes. The first outcome is vitamin A supplementation (VAS) among children aged 6–59 months. Vitamin A supplementation (VAS) coverage was defined as the proportion of eligible children 6–59 months who received at least one dose of vitamin A and expressed as a percentage and frequency (37, 40, 41). The second outcome is the factors associated with V-A supplementation for these children. The association between VAS and associated factors was expressed in the form of Adjusted Odds Ratio (AOR) with their 95% Confidence Intervals (CI). Determinants reported in at least two studies were included in the meta-analysis.

### 2.5 Data extraction

After searching articles from the databases and search engines, three authors (WTW, ABZ, and GBM) screened the titles and abstracts. Articles that passed this initial screening were then eligible for full-text review. The authors (WTW, GBM, EG, BAM, and AAS) independently evaluated the studies for inclusion in the final meta-analysis. Any disagreements among the authors were resolved by consulting the other authors (ANY & GL). Microsoft Excel was used for data extraction. The first author’s last name, year of publication, study area, study design, proportion of the outcome variables, sample size, and adjusted odds ratio with their 95% confidence interval (CI) of the associated factors were extracted from each study. Two independent reviewers (WTW & GBM) extracted the data, and any disagreement between these authors was resolved by re-evaluating the full text and consulting the third author (ABZ) to ensure its accuracy. In cases where data were incomplete, the corresponding authors were contacted twice by email, and if no additional data were provided, calculations were performed based on the available information.

### 2.6 Risk of bias and quality assessment

The methodological quality of each included study was evaluated by three independent authors (WTW, ABZ, and GBM) using the Johana Brigs Institute (JBI) quality appraisal checklist, which was adopted for Cohort and cross-sectional studies (42). The checklist consists of 11 criteria for cohort and 8 criteria for cross-sectional studies. The quality indicators were converted into percentages, and the quality score was graded as high if they scored above 80%, medium quality if they scored between 60 and 80%, and low quality if they scored below 60%. After assessing the quality of each study individually, the three authors met to discuss the quality of each study thoroughly and labeled it based on the JBI scale. Any disagreement that occurs between reviewers was resolved by discussion with a fourth author ANY (Supplementary material 3).

### 2.7 Data synthesis and analysis

After extracting the data in Microsoft Excel, the data was exported to STATA version 17 for further analysis. The pooled estimate of VAS coverage and the effect size of associated factors were estimated using the random effect model with the Restricted Maximum Likelihood ratio (REML) method. Effect sizes were expressed as percentages and Adjusted odds ratio (AOR) with 95% of their Confidence Interval (CI). Heterogeneity across studies was assessed using the Cochrane *Q*-test, tau-squared test, and *I^2^* statistics. To identify the potential source of heterogeneity, a sub-analysis was conducted based on the participants’ age group. Additionally, a sensitivity analysis was done to assess the impact of each study on the pooled VAS estimate by sequentially omitting each study one by one. The small study effect (publication bias) was assessed using a funnel plot (43), and Eggers test (44). The Eggers test result (*p* < 0.005) was used to declare the presence of a small study effect. The association between VAS coverage and associated factors was expressed by the adjusted odds ratio. The results were presented using forest plots and tables.

## 3 Results

### 3.1 Review processes

**Figure 1:**
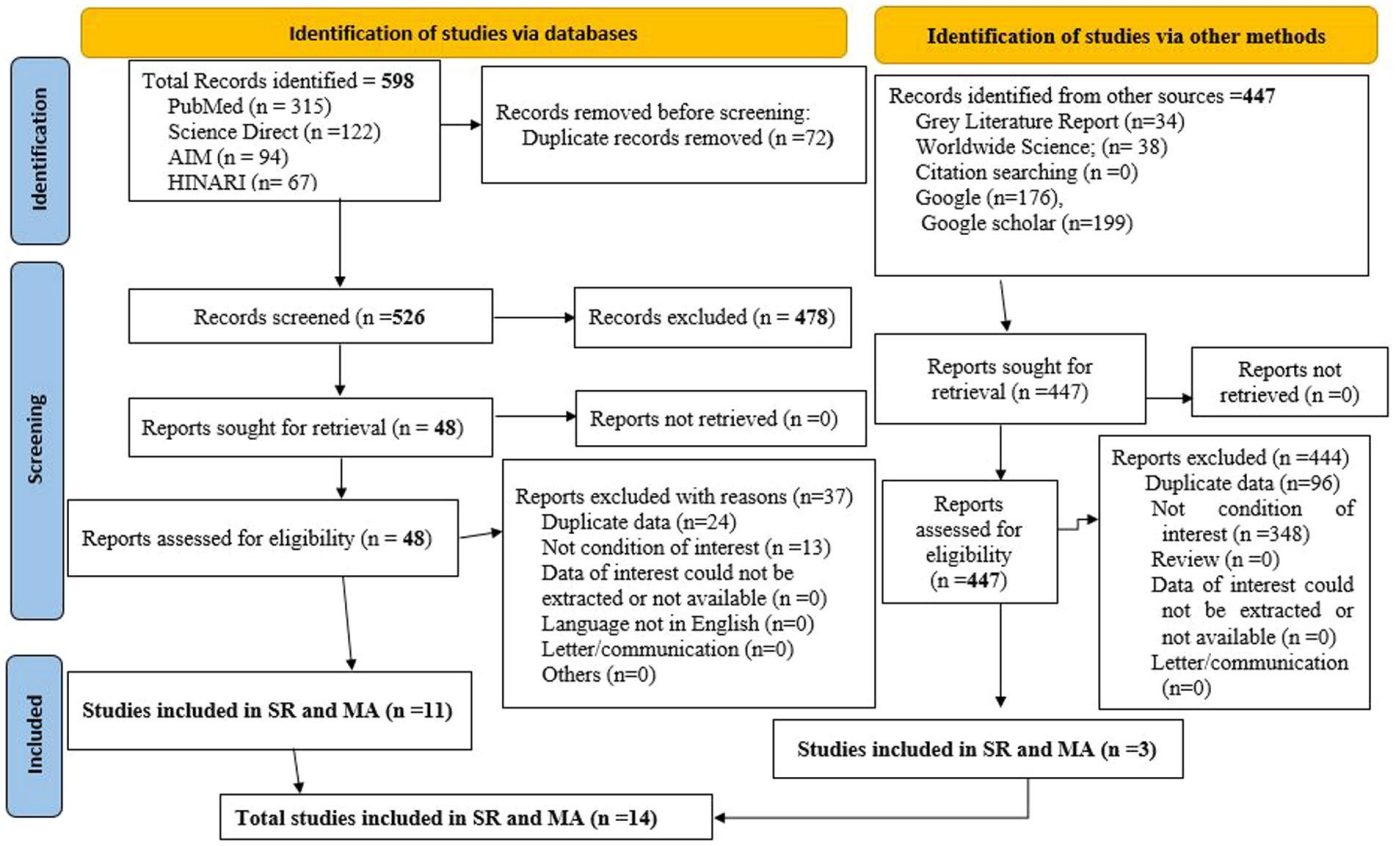
PRISMA 2020 flow diagram showing study searching and selection for vitamin A supplementation coverage and associated factors among children 6–59 months of age in Ethiopia.

Through comprehensive searching, a total of 1,045 articles were found in electronic databases and web search engines. Of these, 598 articles were found in databases (PubMed = 315, Science Direct =122, African Index Medicus (AIM) = 94, HINARI = 67). Out of these 598 studies, 72 were found to be duplicated and removed. The remaining 526 studies were screened by title and abstract for inclusion in the Endnote, among them 478 irrelevant studies were excluded. After removing the irrelevant studies, the remaining 48 studies were screened by full text, out of these 37 studies were excluded based on pre-specified inclusion criteria, and the remaining 11 articles were included in this systematic review and meta-analysis. In the search engines (Google Scholar, Google, World Wide Science) 447 studies were found. Out of these, 444 studies were [duplicated (*n* = 96) and unrelated to the condition of interest (n = 348)]. The remaining three articles were eligible and included in this meta-analysis. Finally, 14 articles (11 from databases and 3 from other search engines) were found to be eligible and included in the final meta-analysis (Figure 1).

### 3.2 Characteristics of studies that report vitamin A supplementation coverage and its associated factors among children 6–59 months of age in Ethiopia

Fourteen studies with a total of 43,047 children aged 6–59 months were found to be eligible and included in this study. Among these studies, the largest sample size was 9,276 (41), while the lowest sample size was 471 (45). The highest coverage of vitamin A supplementation was 83.1% (38) while the lowest VAS was 36.2% (37). Regarding the study region, one study (46) was conducted in Addis Ababa, five studies (37, 38, 45, 47, 48) were in SNNPR, one study (49) was conducted in developed regions of the country (Oromia, Amhara, Tigray Harari and SNNPR), one was conducted in Emerging regions (Afar, Benishangul Gumuz, Gambella, and Somali regions) (50), six studies (41, 51–55) were nationally survey at different time. Regarding the study design 13 (37, 41, 45–55) were cross-sectional and one was a retrospective cohort study (38) (Table 1).

**Table 1:** Characteristics of studies that report vitamin A supplementation coverage and/or its associated factors among children 6–59 months of age in Ethiopia.

ID	Author	Region	Age group	Study design	Sample size	Outcome	Proportion
1	Berihun et al. (45)	SNNP	6–59 months	Cross-sectional	471	273	58
2	Amare et al. (49)	4 regions (Amhara, Oromia, Tigray and SNNP)	6–35 month	Cross-sectional	2,362	1,017	43.06
3	Gebremedhin et al. (38)	SNNP	6–59 months	Retrospective cohort	1,597	1,327	83.1
4	Lucha et al. (51)	Nationally 2019	6–35 months	Cross-sectional	2,242	995	44.4
5	Gebretsadik et al. (46)	Addis Ababa	6–59 months	Cross-sectional	613	365	59.54
6	Kassa et al. (47)	SNNP	6–59 months	Cross-sectional	813	610	75
7	Gilano et al. (52)	Nationally 2016	6–59 months	Cross-sectional	8,973	4,029	44.9
8	Haile et al. (41)	Nationally 2011	6–59 months	Cross-sectional	9,276	4,995	54.5
9	Oumer et al. (54)	Nationally2016	6–59 months	Cross-sectional	8,361	NR	NR
10	Negussie et al. (2021)	SNNP	6–59 months	Cross-sectional	665	241	36.2
11	Gebremedhin al (50)	Emerging regions (Afar, Benishangul Gumuz, Gambella, and Somali)	6–23 months	Cross-sectional	1,009	476	47.2
12	Gambura et, al. (48)	SNNP	6–59 months	Cross-sectional	511	358	70.1
13	Fetene et al. (53)	Nationally2016	6–24 months	Cross-sectional	1,392	708	50.86
14	Richard et al. (2008)	Nationally 2005	12–59 months	Cross-sectional	4,762	2,229	46.8

NR, not reported; SNNPR, South Nation Nationality and Peoples Region.

#### 3.2.1 Quality assessment of the included studies

The included studies’ quality was assessed using the Joana Briggs Institute (JBI) quality assessment checklist for cohort and cross-sectional studies. Accordingly, the included fourteen studies had moderate and good qualities (Supplementary material 3).

### 3.3 Pooled vitamin A supplementation coverage among children 6–59 months of age

**Figure 2:**
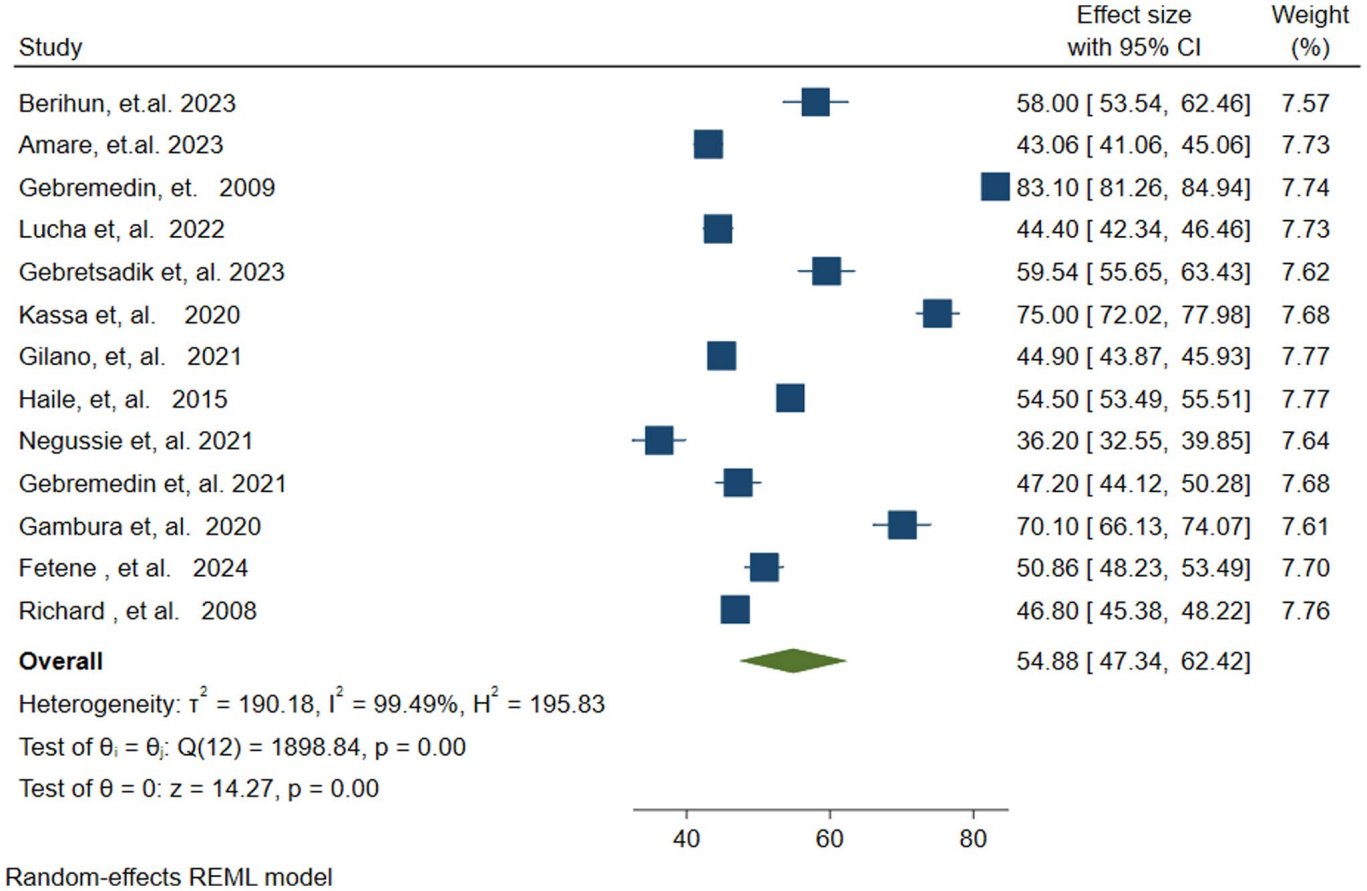
Forest plot showing the pooled vitamin A supplementation coverage among children 6–59 months of age in Ethiopia.

Out of 14 studies, 13 studies (37, 38, 41, 45–53, 55) were included in estimating the pooled vitamin A Supplementation (VAS) Coverage among children in Ethiopia, because one study reported only determinants of VAS. The pooled vitamin A supplementation coverage among children aged 6–59 months in Ethiopia was found to be 54.88% (95% CI: 47.34–62.42). The Random Effects Restricted Maximum Likelihood (REML) model was used to calculate the pooled VAS coverage. In the random effects model, there was significant heterogeneity across the included studies ((Q = 1898 (degree of freedom (df) = 12), *p* = 0.00), Tau-squared = 190.18, H = 195.83, *I^2^* = 99.49%, *p* = 0.00). Hence, to identify the potential source of this heterogeneity a sub-group and sensitivity analysis was conducted (Figure 2).

#### 3.3.1 Investigation of heterogeneity across studies

**Figure 3:**
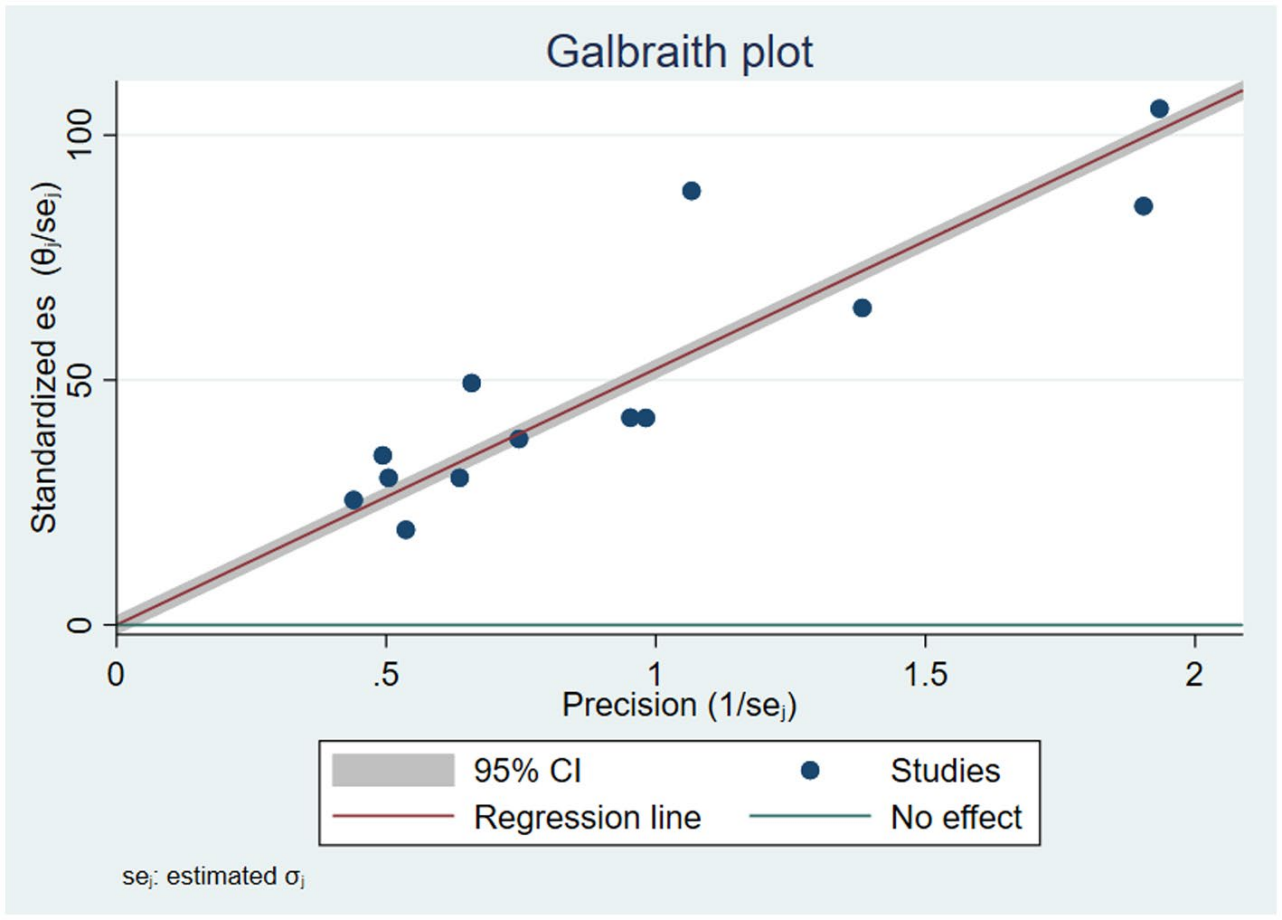
Galbraith plot showing heterogeneity of vitamin A supplementation of children in Ethiopia.

There was a high level of heterogeneity among the included studies in the pooled estimates from the random effects model. This was verified at the statistical value of (Q = 1898, *p* = 0.00, Tau-squared = 190.18, *I^2^* = 99.49%, *p* = 0.00). The heterogeneity was further visualized using forest plots and Galbraith plots. As shown in the Galbraith plot, the majority of the studies were out of the 95% CI (Figure 3). To address and identify the potential source of heterogeneity sub-group analysis, sensitivity analysis, and meta-regression analysis were carried out as outlined below.

#### 3.3.2 Sub-group analysis based on children’s age group

**Figure 4:**
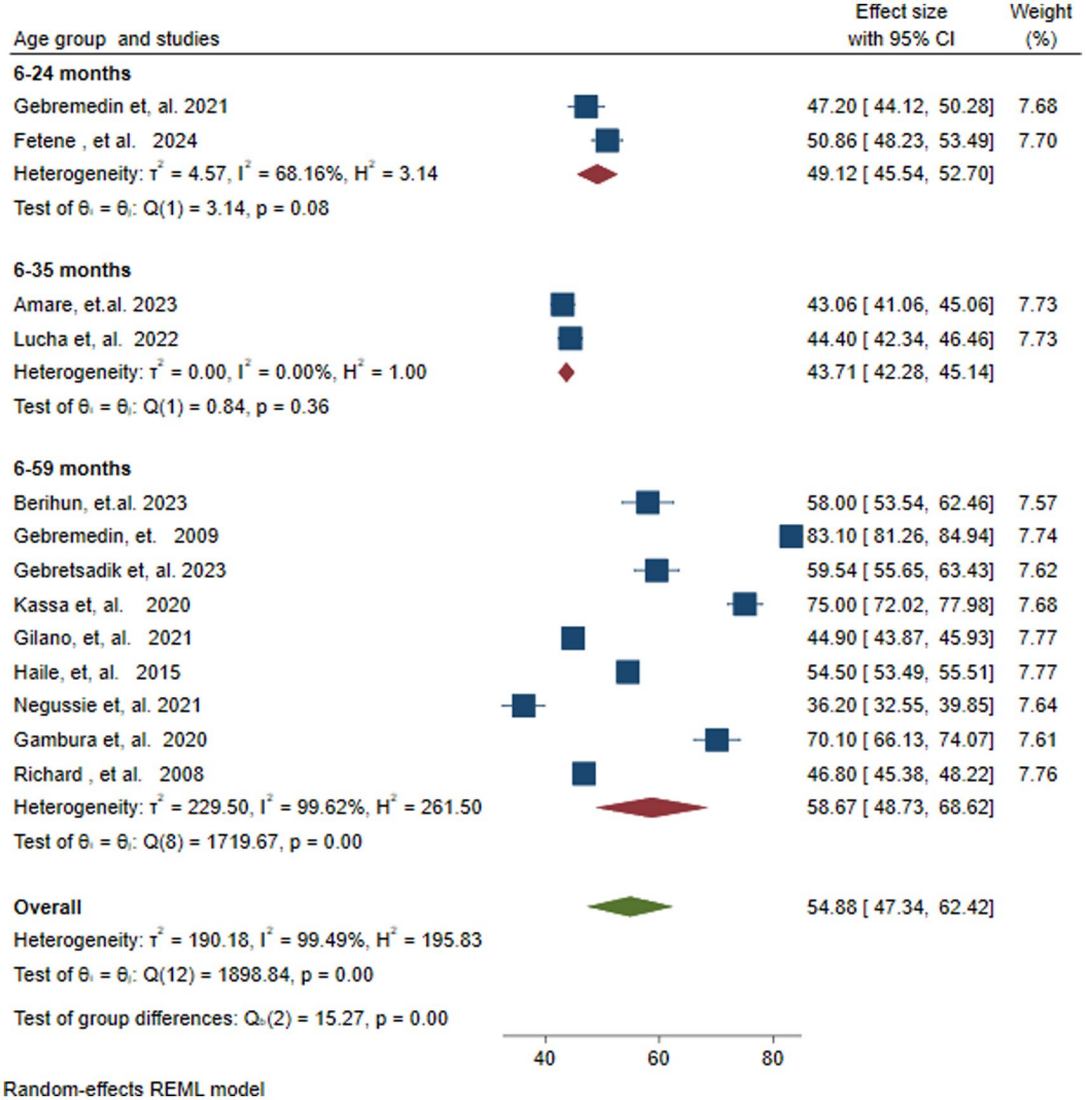
Forest plot showing subgroup analysis of vitamin A supplementation coverage by age group of children in months in Ethiopia.

In the current study, sub-group analysis was performed based on the children’s age group. The lowest VAS coverage of 43.71%% (95% CI: 42.71–45.14) was found in the age group 6–35 months, while the highest VAS coverage of 58.67% (95% CI 48.73–68.62) was found in children 6–59 months of age. The forest plot revealed moderate to substantial heterogeneities among studies reporting VAS coverage for children aged 6–24 months (*I*^2^ = 68.16%. *p* = 0.08) and for those aged 6–59 months (*I*^2^ = 99.49, *p* = 0.00) respectively. However, no heterogeneity was observed in studies that reported VAS coverage for children aged 6–35 months (tau-squared = 0.00, *I*^2^ = 0.00, *p* = 0.36). Additionally, significant heterogeneity was found in the overall pooled estimate (Tau-squared =190.18, *I*^2^ = 99.49, *p* = 0.00) and between groups (test of group differences Q (2) =15.27 and *p* = 0.00) (Figure 4).

#### 3.3.3 Sensitivity analysis

**Figure 5:**
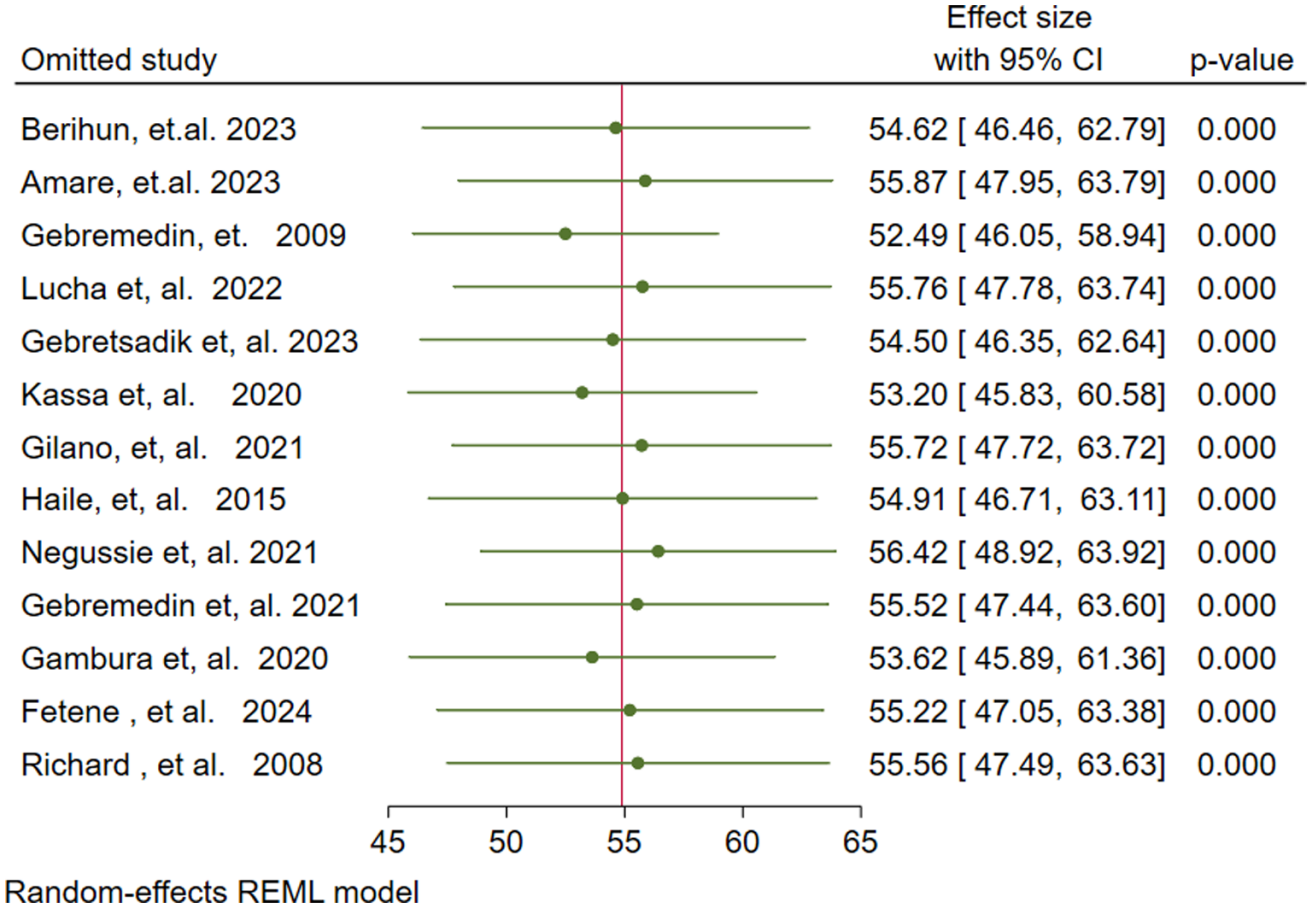
Sensitivity (leave one out) analysis for the vitamin A supplementation coverage among children 6–59 months of age in Ethiopia.

To investigate the presence of a single influential study on the overall pooled estimate of VAS coverage among children aged 6–59 months, a leave-one-out analysis was conducted. The analysis showed that no individual study significantly affected the pooled VAS coverage (54.88%), because all of the single leave-one-out estimates were within the confidence interval of the overall pooled estimate (95% CI: 47.34–62.42) (Figure 5).

#### 3.3.4 Meta-regression analysis

A meta-regression analysis was conducted to explore the source of heterogeneity based on the moderator sample size and publication year. However, none of the moderators were the potential source of variation (Table 2).

**Table 2:** Meta-regression analysis results for VAS among children aged 6–59 months in Ethiopia by publication year and sample size.

The possible source of heterogeneity	Coefficients (95%CI)	Standard error	*P*-value
Publication year	−0.89 (−2.35 to 0.57)	0.75	**0.23**
Sample size	−0.0012 (−0.0037 to 0.0014)	0.0013	**0.363**

VAS:Vitamin A supplementation, CI: Confidence Interval.

#### 3.3.5 Publication bias (small study effect)

**Figure 6:**
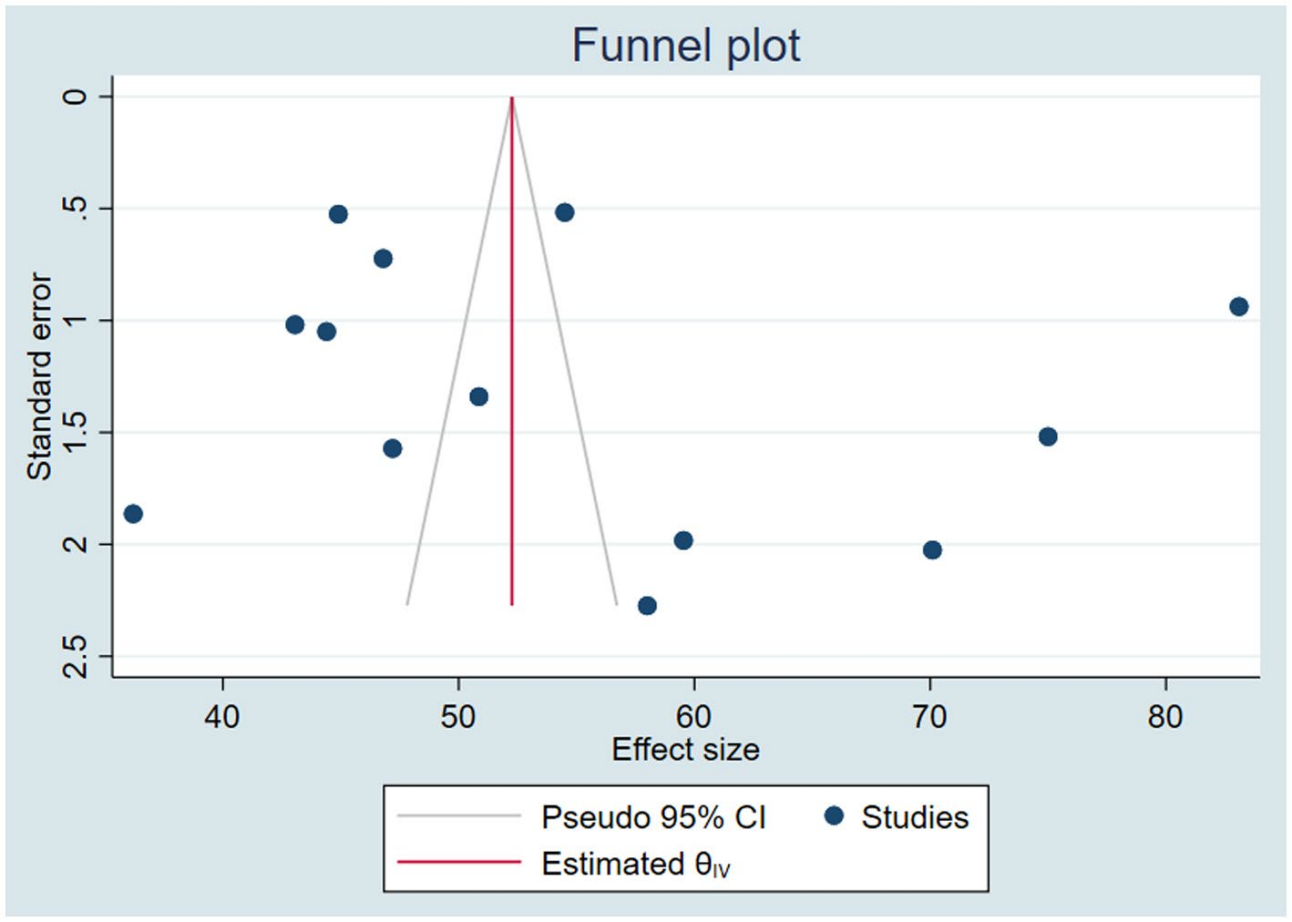
Funnel plot to detect the presence of publication bias/small study effect/ in assessing the pooled VAS coverage and associated factors among children 6–59 months of age in Ethiopia.

The presence of a small study effect (potential publication bias) was assessed both graphically using a funnel plot and statistically through the Eggers test. The funnel plot displayed a symmetrical distribution of the studies, while the Egger test was insignificant (*p* = 0.5534). This implies that there was no evidence of publication bias in the current study (Figure 6).

### 3.4 Factors associated with pooled vitamin A supplementation coverage

A meta-analysis was conducted to identify the pooled effect size of various factors associated with VAS coverage, such as urban resident, antenatal care, birth interval, postnatal care, husbands’ disapproval, mothers’ knowledge, place of delivery, information about VAS, presence of comorbidities, children age, media exposure, sex, time to reach health facilities, mothers educational, occupation, and fathers educational. However, in the pooled estimate, only nine variables were found to be significantly associated with VAS among children as shown below.

#### 3.4.1 Antenatal care

**Figure 7:**
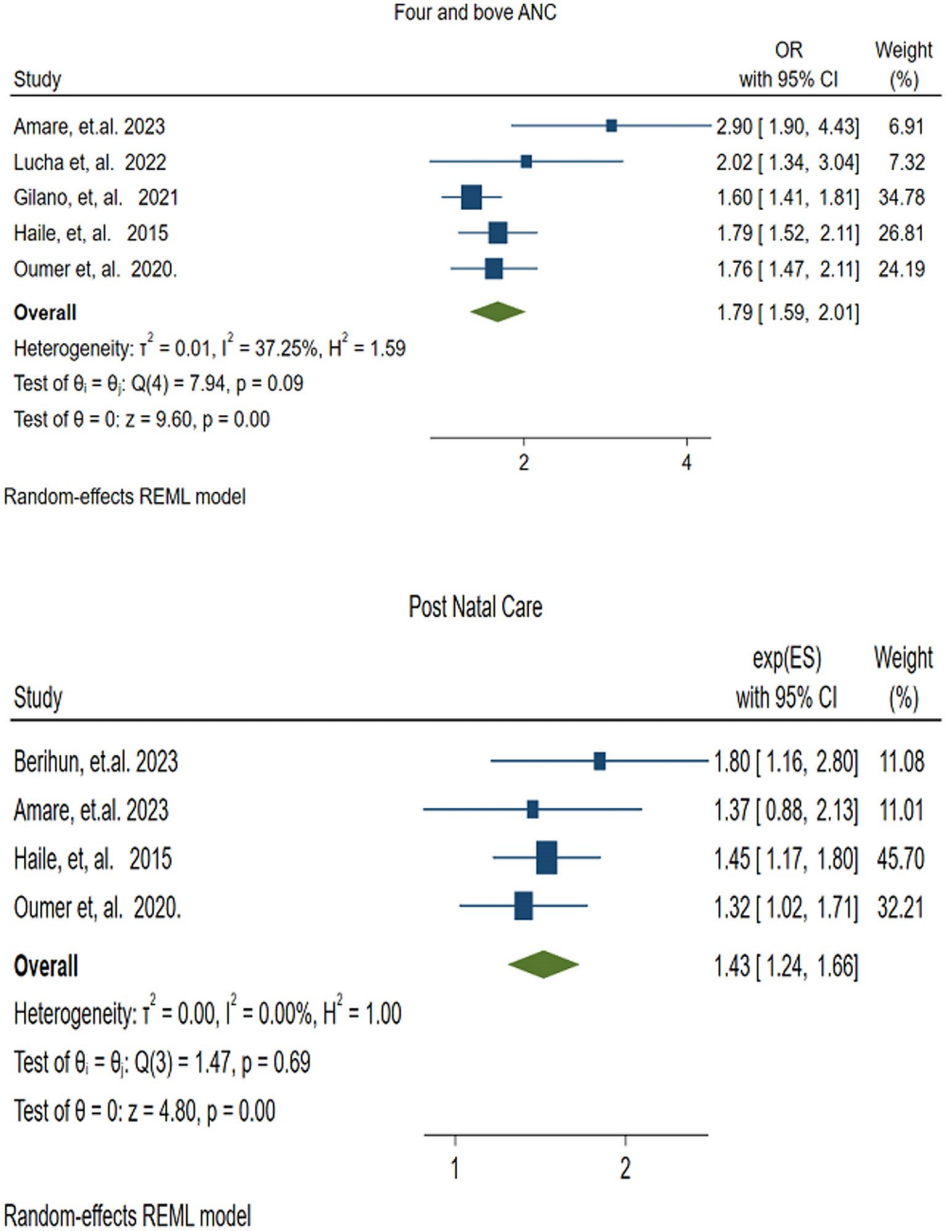
Forest plot showing the association between the factors (four and above antenatal care,and postnatal care), and the pooled vitamin A supplementation among children 6–59 months of age in Ethiopia.

Five studies (41, 49, 51, 52, 54) reported the association between VAS and Antenatal Care (ANC) of four or more visits. The pooled analysis revealed that children of mothers who had four and more ANC visits were nearly twice (OR = 1.79, 95% CI: 1.59–2.01) as likely to receive vitamin A compared with children of mothers with fewer than four ANC visits. There was no statistically significant heterogeneity among the included studies (*I*^2^ *=* 37.25, *p* =0.09) (Figure 7).

#### 3.4.2 Postnatal care

Four studies (41, 45, 49, 54) were included to assess the association between VAS and Postnatal Care (PNC). The random effects model revealed that mothers who received PNC were 1.43 times more likely to receive vitamin A for their children than those mothers who did not receive PNC (OR = 1.43 (95% CI: 1.24–1.66)). There was no variation among studies (*I^2^* = 0.00, *p* = 0.69) (Figure 7).

#### 3.4.3 Delivery at a health facility

**Figure 8:**
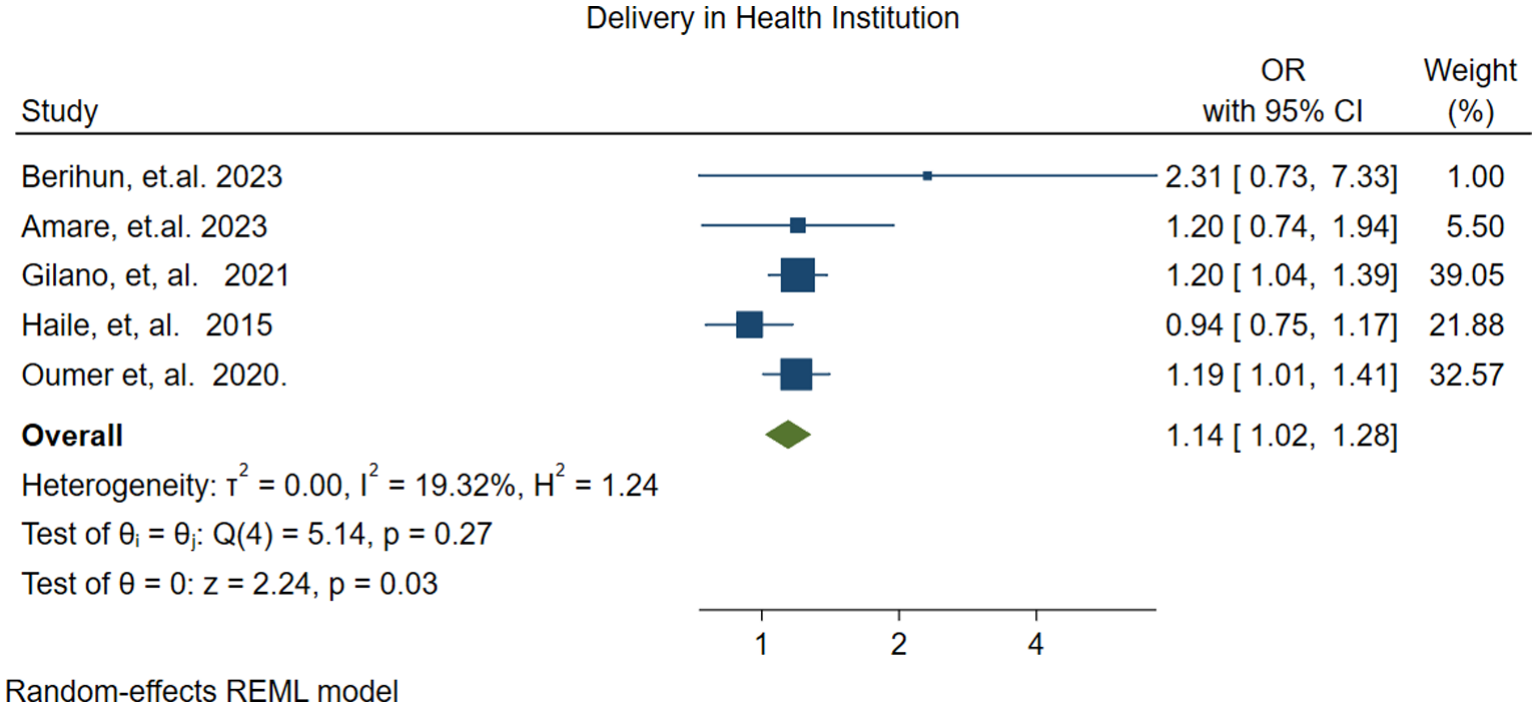
Forest plot showing the association between delivery at health facility and vitamin A supplementation among children 6–59 months of age in Ethiopia.

The pooled effect of delivery at a health facility on vitamin A supplementation was assessed using five studies (41, 45, 49, 52, 54). The pooled effect size revealed that children born in health facilities were 1.14 times more likely (OR = 1.14, 95% CI: 1.02–1.28) to receive vitamin A supplementation compared to those children born outside health facilities. There was no statistically significant variability among the studies (*I*^2^ = 19.32%, *p* = 0.27) (Figure 8).

#### 3.4.4 Parents media exposure

**Figure 9:**
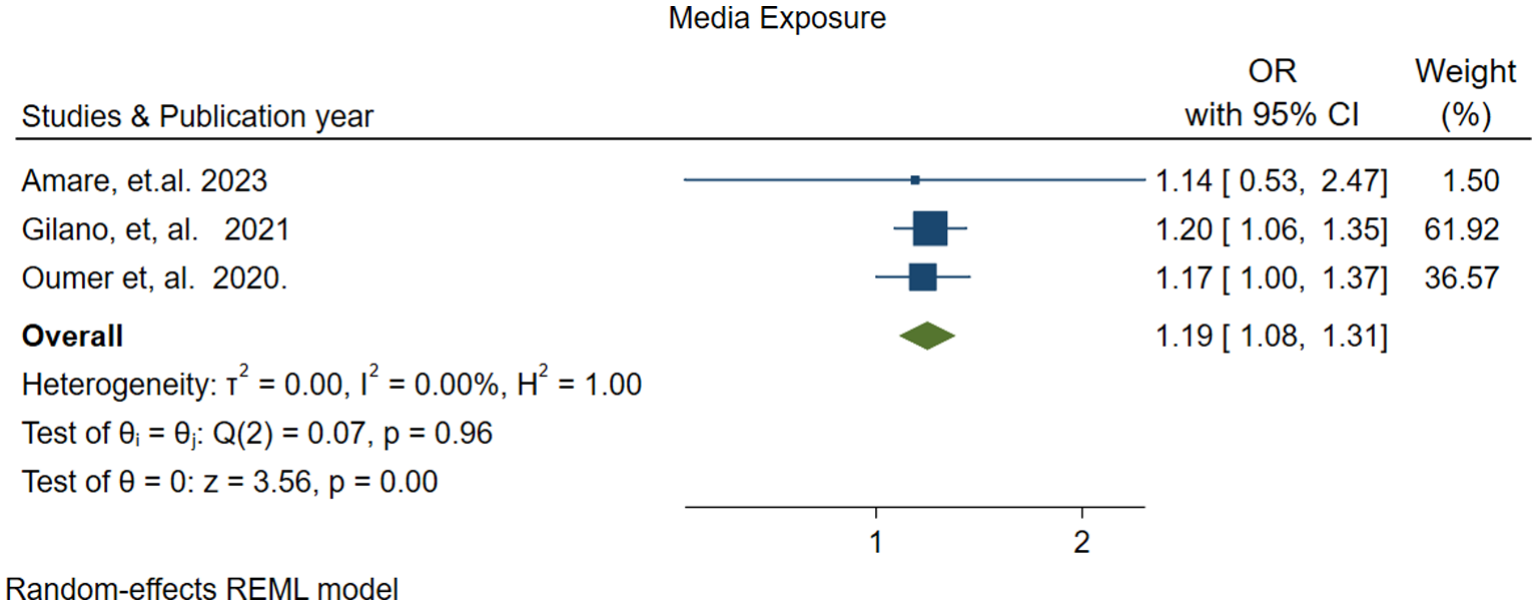
Forest plot showing the association between parents’ media exposure and vitamin A supplementation among children 6–59 months of age in Ethiopia.

The association between parental media exposure and vitamin A supplementation (VAS) was reported in three studies (49, 52, 54). The pooled analysis indicated that parental media exposure increased the likelihood of children receiving vitamin A by 1.19 times [OR: 1.19 (95% CI; 1.08–1.31)] compared to those whose parents were not exposed. There was no heterogeneity (*I*^2^ = 0.00%, *p* = 0.96) (Figure 9).

#### 3.4.5 Time taken to reach health facilities

**Figure 10:**
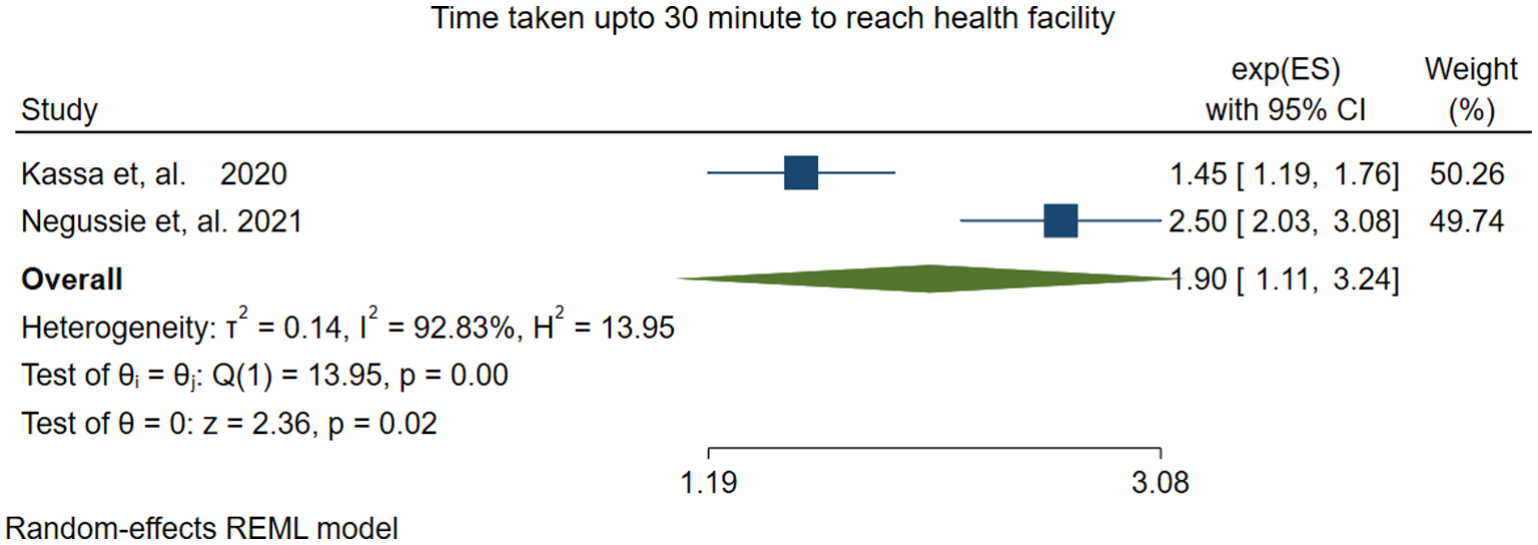
Forest plot showing the association between time taken to reach health facilities within 30 min and VAS among children 6–59 months of age in Ethiopia.

Two studies (37, 47) reported the association between VAS and the time it takes to reach a health facility. The pooled result revealed that children/parents who reached the nearest health facility within 30 min were twice as likely to receive vitamin A compared to those who took longer part [OR: 1.90, (95% CI: 1.11–3.24)]. The random effects model revealed substantial heterogeneity between studies (*I^2^* = 92.83, *p* = −0.00). Due to the small no of studies, we did not assess publication bias (Figure 10.)

#### 3.4.6 Parents’ information about VAS

**Figure 11:**
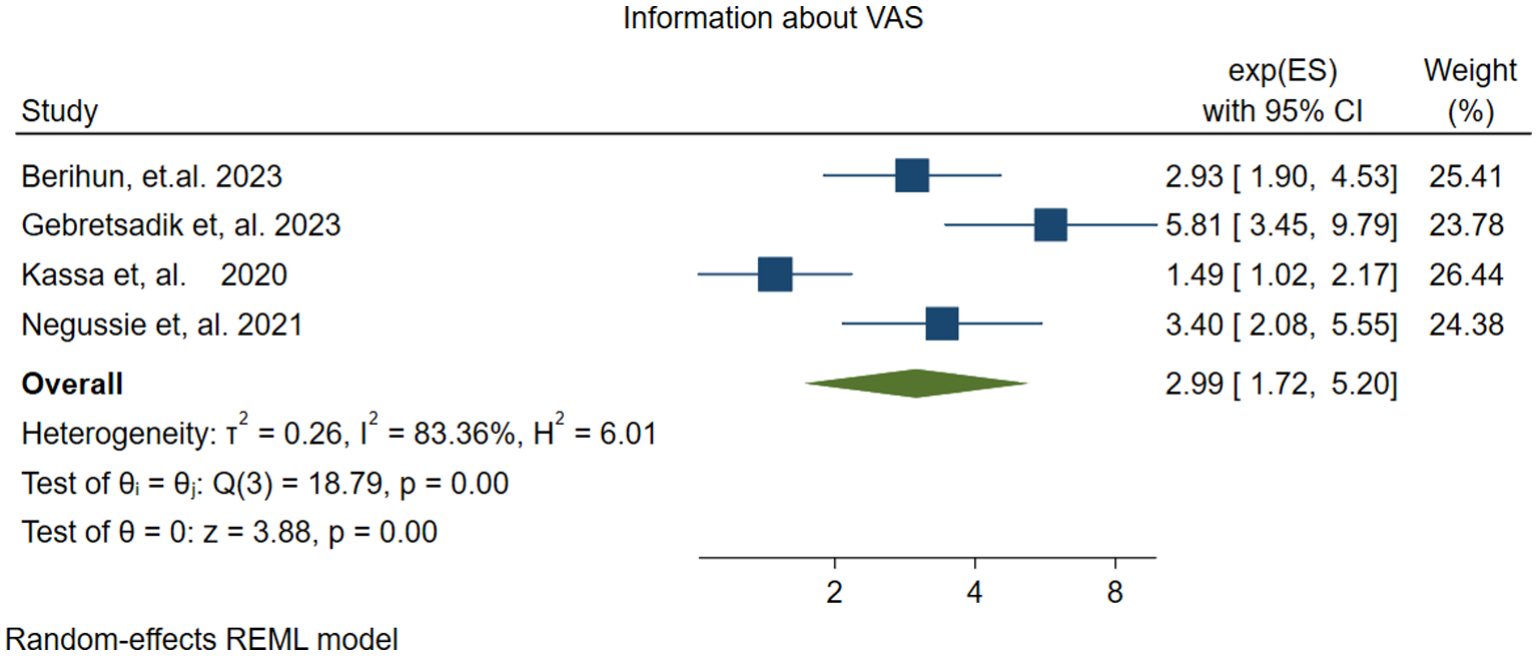
Forest plot showing the association between parents having information about vitamin A supplementation and vitamin A supplementation among children 6–59 months.

Four studies (37, 45–47) investigated the association between vitamin A supplementation (VAS) in Children and having parents’ information about VAS. The random effects model revealed that children whose parents had information about VAS were three times more likely to receive it than those whose parents lacked such information (OR = 2.99; 95% CI: 1.72–5.20). The pooled estimate showed significant heterogeneity (*I*^2^ = 83.36, *P=*− 0.00) (Figure 11).

#### 3.4.7 Maternal secondary educational level

**Figure 12:**
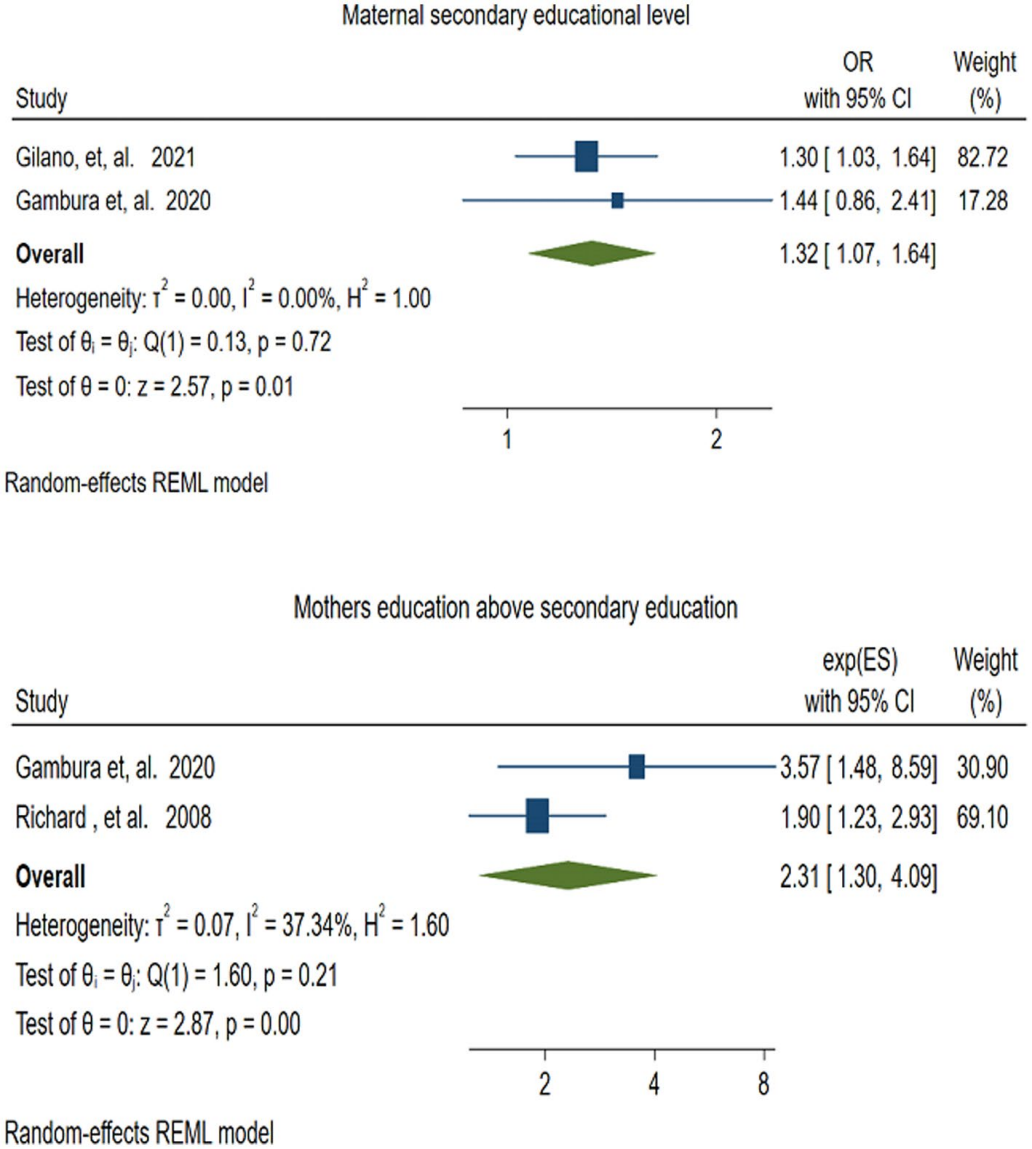
Forest plot for depicting the association between maternal secondary educational level and educational level of above secondary school, and VAS among children 6–59 months.

To evaluate the effect of mothers’ secondary educational level on vitamin A supplementation in children, two studies (48, 52) were included. The pooled effect size revealed that children of mothers with secondary educational levels were 1.32 times more likely to receive VAS compared to those children whose mothers were illiterate (OR = 1.32; 95% CI: 1.07–1.64). In this meta-analysis, there was no evidence of heterogeneity among studies (*I^2^* = 0.00, *P* = −0.72) (Figure 12).

#### 3.4.8 Mothers’ education above secondary education

Two studies (48, 55) assessed the association between vitamin A supplementation (VAS) and mothers’ education beyond secondary school. The pooled result depicted that, children of mothers with education above secondary school were more than twice as likely to receive VAS compared to their counterparts (OR = 2.31; 95% CI: 1.30–4.09). There was no statistically significant heterogeneity among the included studies (*I^2^* = 37.34, *p* = −0.20) (Figure 12).

#### 3.4.9 Father’s educational level

**Figure 13:**
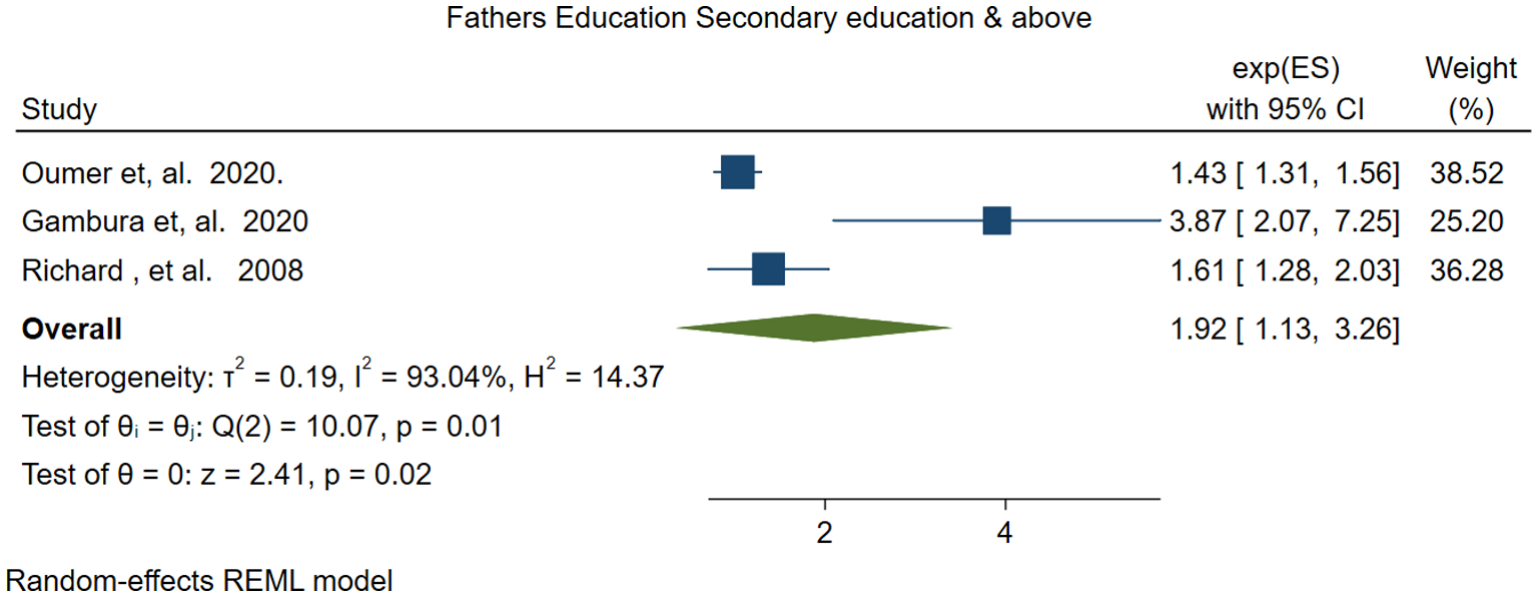
Forest plot for depicting the association between fathers’ secondary educational level or above, and vitamin A supplementation among children 6–59 months.

Three studies (48, 54, 55) were used to examine the association between fathers’ educational level and VAS coverage among children aged 6–59 months in Ethiopia. The meta-analysis results indicated that children whose fathers had an educational level above secondary school were twice as likely to receive VAS compared to children of illiterate fathers (OR = 1.92; 95% CI: 1.13–3.26). The heterogeneity test showed substantial heterogeneity (*I^2^* = 93.04, *p =* −0.01) (Figure 13).

#### 3.4.10 Summary of findings

In summary, the study identified several statistically significant associated factors of vitamin A Supplementation, including antenatal care, postnatal care, delivery in a health institution, parents’ media exposure, a travel time of 30 min or less to reach the health facility, Parents having information about VAS, maternal secondary educational level, mother educational level above secondary school, fathers educational level above secondary school (Table 3).

**Table 3:** Summary of associated factors of vitamin A Supplementation among children aged 6–59 months in Ethiopia.

Associated factors	No of studies	PAOR (95% CI)	Heterogeneity test	
			I2	*P*-value
Antenatal care	5	1.79 (1.59–2.01)	37.25	0.09
Post-natal care	4	1,43 (1.24–1.66)	0.00	0.69
Delivery in a health institution	5	1.14 (1.02–1.28)	19.32	0.27
Parents media exposure	3	1.19 (1.08–1.31)	0.00	0.96
Time taken 30 min to reach the health facility	2	1.90 (1.11–3.24)	92.83	0.00
Parents having information about VAS	4	2.99 (1.72–5.20)	83.36	0.00
Maternal secondary educational level	2	1.32 (1.07–1.64)	0.00	0.72
Mother’s educational level above secondary education	2	2.31 (1.31–4.09)	37.34	0.20
Father’s educational level above secondary education	3	1.92 (1.13–3.26)	93.04	0.01

PAOR, pooled adjusted odds ratio.

## 4 Discussion

This meta-analysis aimed to assess the coverage of vitamin A supplementation and its associated factors among children 6–59 months of age in Ethiopia. The pooled coverage of vitamin A supplementation was found to be 54.88% (95% CI: 47.34–62.42). The current study finding was consistent with studies conducted in Tanzania (53.18%) (18), India (60.5%) (20), East and South Africa (57%) (12), and Sub-Saharan African countries (56.3%) (13). The similarity in findings may be attributed to comparable socio-demographic and economic characteristics of the study participants, study settings, and maternal and child health care services.

In contrast, the finding of this study was higher than those reported in Mozambique (42.8%), Senegal (46.1%), Cambodia (42.8%) (19), and Brazil (41.5%) (21). However, it was lower than studies conducted in West and Central Africa (71%) (12), Sierra Leone (86.9%) (14), Guinea (68%) (15), Mali (80%) (16), and South Asia (62%) (12). This disparity could be attributed to differences in the methods of vitamin A supplementation (VAS). In these counties, VAS is provided not only through routine immunization programs but also through the annual VAS campaign and national immunization day. As a result, children in these countries have better access to VAS compared to those in Ethiopia, where it is primarily distributed through the routine immunization program. Another contributing factor could be the intensive outreach efforts in those countries, which effectively target disadvantaged and older children who may fall outside the routine immunization schedule.

Additionally, these countries may have better infrastructures, such as well-developed roads and nearby health facilities, which makes VAS more accessible. In contrast, in Ethiopia, inadequate infrastructure, particularly in rural and remote areas, hinders access to vitamin A, which contributes to these disparities (56).

In this study, a sub-group analysis was conducted based on the Children’s age group to identify the possible source of heterogeneity. The lowest VAS coverage [43.71%% (95% CI: 42.71–45.14)], was found among children 6–35 months of age. This may be due to the parents’ perception that children in this age group receive enough nutrition, including vitamin A, through breast milk. Additionally, parents may be more aware of the benefit of vitamin A for older children, leading to less proactive supplementation efforts for children aged 6–35 months. To improve VAS coverage in this age group, targeted outreach supplementation, raising caregiver awareness, and integration of health services are significantly necessary and need to be prioritized (57).

This study also examined the associated factors of VAS. This study finding revealed that children whose mothers had attended four or more ANC visits were twice as likely to receive VAS. This study finding was aligned with studies conducted in Nigeria (17), and Tanzania (18). A possible explanation is that mothers who had ANC visits receive information about child vaccinations and the nutritional importance of vitamin A. As a result, they are more likely to take their children to health facilities and campaign sites for supplementation (58). This study also found that being able to reach a health facility within 30 min was an important factor associated with vitamin A supplementation in children. Similar findings have been reported in Kenya (59), and Malawi (60). One reason could be that as the distance or travel time increases, parents may be less inclined to make the journey to obtain vitamin A for their children. Another reason could be that the cost and time required for transportation place a significant burden on families, especially those living in poverty (61). Furthermore, underdeveloped and poor road infrastructures, particularly, in rural areas, affect access to health facilities and routine services (62).

The current study identified Postnatal Care (PNC), as a determinant of vitamin A supplementation in children. This finding is supported by a systematic review and meta-analysis conducted in Asia and Africa (63). One possible reason is that during postnatal visits, healthcare workers educate caregivers on the importance of vitamin A supplementation for child health, growth, and development (64). Additionally, PNC offers an opportunity for growth monitoring and assessment of children’s nutritional status (65), facilitating the early detection of vitamin A deficiency and the timely provision of high-dose vitamin A supplementation to address the deficiency.

Delivery in a health facility was another significant factor influencing VAS for children in this study. Congruent findings were reported in Nigeria (17), and Tanzania (18). This can be attributed to the fact that newborns delivered in health facilities are linked to routine immunization and other child health services, such as growth monitoring, which helps to create a follow-up visit. Consequently, these children are more likely to receive subsequent doses of vitamin A supplements at the appropriate ages and intervals. Another possible reason is that health facilities collaborate with health extension workers to improve child immunization coverage. They organize outreach immunization campaigns, including vitamin A supplementation for children 6–59 months, which increases the likelihood of children receiving vitamin A supplementation, even if they miss a dose at the health facility (66). In agreement with studies from Sub-Saharan Africa (22), Mali (16), Tanzania (18), Bangladesh (25), and other Low and Middle-Income Countries (LIMIC) (26), this study also found that parents/caregivers media exposure was a significant factor in children vitamin A supplementation. This is because media exposure educates the parents/caregivers about the importance of immunization in protecting against communicable diseases. Another possible reason is that the media provides up-to-date information about vitamin A, its role in disease prevention, and its dietary sources, helping to raise awareness and dispel common myths and fears within the community (67). Additionally, media interviews with health professional can also provide authoritative and timely responses to the parents’ questions. Furthermore, social media platforms can highlight the risks of not vaccinating and provide evidence-based information, which encourages parents to take actions (68).

This study further showed that children whose parents had information about VAS were three times more likely to receive it. This finding aligns with studies conducted in Kenya (28), and Sub-Saharan African countries (30). A likely explanation is that parents who are informed about vitamin A’s role in preventing night blindness and overall health are more aware, and proactive in seeking vitamin A supplementation for their children. Hence this finding suggests that strengthening the VAS program through clear information and good communication can increase parents’ health-seeking behavior and improve VAS utilization (69).

Mothers’ secondary education was a significant determinant for supplementing vitamin A in Children. A consistent finding was reported from Nigeria (17, 21), Bangladesh (25), and Cambodia (19). Additionally, maternal education above secondary school is also associated with vitamin A supplementation. This is consistent with studies from Kenya (28), Sub-Saharan Africa (23), and India (29). This may be because educated mothers have a better understanding of health and nutrition including the role of vitamin A in preventing night blindness. Another reason could be that educated mothers are more proactive in seeking healthcare services and information for their children. They are more likely to take their children for regular check-ups and ensure they receive the recommended vitamin A supplements at the appropriate times (70). In addition, to the reasons, aforementioned earlier, educated mothers share information about the importance of VAS through social media and peers, participate in community-based health programs, and have a strong voice in household decisions, which creates a supportive environment for VAS (71).

This study also found that fathers’ education had a significant association with VAS in Children. This finding is consistent with similar studies conducted in Bangladesh (25, 27), and Mali (16). A possible reason could be fathers with higher educational levels are more likely to understand the value of health services like VAS and make decisions to ensure their children receive VAS (72). Another possible reason is that educated fathers tend to have better health-seeking behaviors and emphasize the importance of preventive health practices like VAS to the mother and caregiver, which creates an opportunity for their children to receive supplementation (73).

## 5 Strengths and limitations

This is the first review that showed the pooled coverage of vitamin A supplementation and factors associated with it at the national level. However, all of the included studies were cross-sectional studies, by nature which may not show real temporal relationships between outcome and independent variables. Additionally, certain factors like the wealth index of the family and maternal age were not assessed, because the primary studies categorized these variables inconsistently.

## 6 Conclusion and recommendations

In Ethiopia, the vitamin A supplementation coverage is significantly lower than both the WHO recommendation of 80% and the national target of 95%. Antenatal care, postnatal care, delivery in health facilities, Parents’ media exposure, time to reach health facilities (less than 30 min), maternal secondary educational level or above, and father’s education above secondary school were significantly associated factors that increase the likelihood of vitamin A supplementation.

Hence, the National nutritional program and expanded program of immunization stakeholders better to increase awareness of the community about VAS and other routine immunizations, particularly for parents/caregivers with low educational status, and no antenatal and postnatal care through social media and other community meetings. Additionally, like other Sub-Saharan African countries, Ethiopia’s EPI program should strengthen the outreach supplementation programs, such as door-to-door distribution to address the losses of older children and socio-economically disadvantaged populations in remote and rural areas.

## Data Availability

The original contributions presented in the study are included in the article/Supplementary material, further inquiries can be directed to the corresponding author.
