# Supplementary material for: Vitamin A supplementation coverage and its associated factors among children 6–59 months of age in Ethiopia: a systematic review and meta-analysis
Source: Front Public Health. 2025 Apr 8;13:1496931. doi: 10.3389/fpubh.2025.1496931 (PMC12011748; doi:10.3389/fpubh.2025.1496931)
Supplement: Supplementary file 2 [file Table_2.DOCX]

Coverage and associated factors of Vitamin A supplementation among children aged 6-59 months in Ethiopia: A systematic review and meta-analysis.

Additional file Table 2: Search terms and strategy option for data bases (PubMed, African Index Medicus (AID), Science direct, and HINARI) and web search engines (Google, Google scholar and worldwide Science**)**

|  | Concept 1 | Concept 2 | Concept 3 | Concept 5 | Concept 6 |
| --- | --- | --- | --- | --- | --- |
| Free text word | Coverage | “Associated factors” | “Vitamin-A supplementation” | “Children 6-59 months” | Ethiopia |
| Mesh | "Vaccination Coverage"[Mesh] | “Precipitating Factors"[Mesh] | "vitamin a"[MeSH Terms] | "child"[MeSH Terms] | - |
| Related Terms | Prevalence  Magnitude  Proportion  Uptake  Differential  “Micro-nutrient intake”  Estimates | Determinants  Epidemiologic factors  Predictors  “Risk factors”  Barriers  Facilitators | Vitamin A*  Retinol  “Pro vitamin A”  “Vitamin A Distribution”  “Vitamin A Provision “  Vitamin A Supplement*  “Dietary supplement” | Child*  Infants*  “Preschool children”  Toddler*  “Under five years’ children”  Children “6-36 months”  “Children 6-24 months” |  |

**Pub Med=315**

((((((((((Coverage[Title/Abstract]) OR ("Vaccination Coverage"[Mesh])) OR (Prevalence[Title/Abstract])) OR (Magnitude[Title/Abstract])) OR (Proportion[Title/Abstract])) OR (Uptake[Title/Abstract])) OR (Differential[Title/Abstract])) OR ("Micro-nutrient intake"[Title/Abstract])) AND ((((((((("Associated factors"[Title/Abstract]) ) OR ("Precipitating Factors"[Mesh])) OR (Determinants[Title/Abstract])) OR ("Epidemiologic factors"[Title/Abstract])) OR (Predictors[Title/Abstract])) OR ("Risk factors"[Title/Abstract])) OR (Barriers[Title/Abstract])) OR (Facilitators[Title/Abstract]))) AND ((((((("Vitamin A supplementation"[Title/Abstract]) OR ("vitamin a"[MeSH Terms])) OR (Vitamin A[Title/Abstract])) OR ("Retinol supplementation"[Title/Abstract])) OR ("Pro vitamin A "[Title/Abstract])) OR (" Vitamin A Supplement*"[Title/Abstract])) OR ("Dietary supplementation"[Title/Abstract]))) AND (((((((("Children 6-59 months"[Title/Abstract]) OR ("child"[MeSH Terms])) OR (Child*[Title/Abstract])) OR (Infants*[Title/Abstract])) OR ("Preschool children"[Title/Abstract])) OR ("Under five years’ children"[Title/Abstract])) OR ("Children 6-36 months"[Title/Abstract])) OR ("Children 6-24 months"[Title/Abstract])) ND (Ethiopia[Title/Abstract])

**HINARI n= 67**

(Coverage OR Prevalence OR Differential OR Magnitude OR Proportion OR Uptake) AND (Associated factors OR Determinants OR Predictors OR Barriers) AND (“Vitamin A supplementation” OR “Pro vitamin A” OR Provision OR “Vitamin A Supplement*) AND (Child* OR “Preschool children” OR Children 6- 59 months OR children OR 6-36 months OR 6-24 months) AND (Ethiopia)

**African Index Medicus (AIM) = 94)**

(Coverage OR Prevalence OR Magnitude OR Proportion OR Uptake OR Differential) AND (Associated factors OR Determinants OR Predictors OR “Risk factors” OR Barriers) AND (Vitamin A* OR “Pro vitamin A” OR “Vitamin A Supplement*) AND (children)

**Science direct n=122**

(Coverage OR Magnitude OR Uptake) AND (“Associated factors” OR Determinants) AND (“Vitamin-A supplementation”) AND (Children OR “Children 6-59 months”) AND (Ethiopia)

**Google Scholar (N=199)**

(Coverage OR Prevalence OR Magnitude OR Uptake OR Differential OR “Micro-nutrient intake") AND (“Associated factors” OR Determinants OR Epidemiologic factors OR Predictors OR “Risk factors” OR Barriers) AND (“Vitamin A supplementation” OR Vitamin A* ) AND (“Children 6-59 months” Child* OR “Preschool children” OR “Under five years’ children”) AND (Ethiopia)"

**Google (N=176)**

(Coverage OR Magnitude OR Uptake) AND (“Associated factors” OR Determinants OR Barriers) AND (“Vitamin A supplementation” OR Vitamin A* ) AND (“Children 6-59 months” Child*) AND (Ethiopia)"

**Worldwide Science (n= 72)**

(Coverage OR Magnitude OR Uptake) AND (“Associated factors” OR Determinants OR Barriers) AND (“Vitamin A supplementation” OR Vitamin A* ) AND (“Children 6-59 months” Child*) AND (Ethiopia)"
