# Supplementary material for: Vitamin A supplementation coverage and its associated factors among children 6–59 months of age in Ethiopia: a systematic review and meta-analysis
Source: Front Public Health. 2025 Apr 8;13:1496931. doi: 10.3389/fpubh.2025.1496931 (PMC12011748; doi:10.3389/fpubh.2025.1496931)
Supplement: Supplementary file 3 [file Table_3.DOCX]

/Additional file Table 3 A: Quality assessment of included Cross-sectional studies using JBI Quality appraisal checklist.

| Author, Year | Q1 | | | Q2 | | | | Q3 | | | | Q4 | | | | Q5 | | | | Q6 | | | | Q7 | | | | Q8 | | | | Overall quality in % |
| --- | --- | --- | --- | --- | --- | --- | --- | --- | --- | --- | --- | --- | --- | --- | --- | --- | --- | --- | --- | --- | --- | --- | --- | --- | --- | --- | --- | --- | --- | --- | --- | --- |
|  | N | U | NA | Y | N | U | NA | Y | N | U | NA | Y | N | U | NA | Y | N | U | NA | Y | N | U | NA | Y | N | U | NA | Y | N | U | NA |  |
| Berihun, et al, 2023. | **√** |  |  | **√** |  |  |  | **√** |  |  |  | **√** |  |  |  | **√** |  |  |  | **√** |  |  |  | **√** |  |  |  | **√** |  |  |  | 8/8 (100) |
| Amare, et al, 2023. | **√** |  |  | **√** |  |  |  | **√** |  |  |  | **√** |  |  |  | **√** |  |  |  | **√** |  |  |  | **√** |  |  |  | **√** |  |  |  | 8/8 (100) |
| Lucha ,et al, 2022. | **√** |  |  | **√** |  |  |  | **√** |  |  |  |  |  | **√** |  | **√** |  |  |  | **√** |  |  |  | **√** |  |  |  | **√** |  |  |  | 7/8 (87.5) |
| GebretsadiK , et.al, 2023. | **√** |  |  | **√** |  |  |  | **√** |  |  |  | **√** |  |  |  | **√** |  |  |  | **√** |  |  |  | **√** |  |  |  | **√** |  |  |  | 8/8 (100) |
| Kassa, et al, 2020 | **√** |  |  | **√** |  |  |  | **√** |  |  |  | **√** |  |  |  | **√** |  |  |  | **√** |  |  |  | **√** |  |  |  | **√** |  |  |  | 8/8 (100) |
| Gilano, et.al, 2021 | **√** |  |  | **√** |  |  |  | **√** |  |  |  | **√** |  |  |  | **√** |  |  |  | **√** |  |  |  | **√** |  |  |  | **√** |  |  |  | 8/8 (100) |
| Haile, et al, 2015. | **√** |  |  | **√** |  |  |  | **√** |  |  |  | **√** |  |  |  | **√** |  |  |  | **√** |  |  |  | **√** |  |  |  | **√** |  |  |  | 8/8 (100) |
| Oumer et al, 2020. | **√** |  |  | **√** |  |  |  |  | **√** |  |  | **√** |  |  |  | **√** |  |  |  | **√** |  |  |  | **√** |  |  |  | **√** |  |  |  | 7/8 (87.5) |
| Negussie, et al, 2021. | **√** |  |  | **√** |  |  |  | **√** |  |  |  | **√** |  |  |  |  | **√** |  |  | **√** |  |  |  | **√** |  |  |  | **√** |  |  |  | 7/8(87.5) |
| Gebremedin et al, 2021 | **√** |  |  | **√** |  |  |  | **√** |  |  |  | **√** |  |  |  |  | **√** |  |  |  | **√** |  |  | **√** |  |  |  | **√** |  |  |  | 6/8 (75) |
| Gambura et al, 2020 | **√** |  |  | **√** |  |  |  | **√** |  |  |  | **√** |  |  |  | **√** |  |  |  | **√** |  |  |  | **√** |  |  |  | **√** |  |  |  | 8/8 (100) |
| Fetene, et al, 2024. | **√** |  |  | **√** |  |  |  | **√** |  |  |  | **√** |  |  |  |  | **√** |  |  |  | **√** |  |  | **√** |  |  |  | **√** |  |  |  | 6/8 (75) |
| Richard , et al, 2008. | **√** |  |  | **√** |  |  |  | **√** |  |  |  | **√** |  |  |  | **√** |  |  |  | **√** |  |  |  | **√** |  |  |  | **√** |  |  |  | 8/8 (100) |

Additional file Table 3 B: Quality assessment of included cohort studies in meta-analysis for Cohort study

| Author, Year | Q1 | | | | Q2 | | | | Q3 | | | | Q4 | | | | Q5 | | | | Q6 | | | | Q7 | | | | Q8 | | | | Q9 | | | | Q10 | | | | Q11 | | | | Overall quality |
| --- | --- | --- | --- | --- | --- | --- | --- | --- | --- | --- | --- | --- | --- | --- | --- | --- | --- | --- | --- | --- | --- | --- | --- | --- | --- | --- | --- | --- | --- | --- | --- | --- | --- | --- | --- | --- | --- | --- | --- | --- | --- | --- | --- | --- | --- |
|  | Y | N | U | NA | Y | N | U | NA | Y | N | U | NA | Y | N | U | NA | Y | N | U | NA | Y | N | U | NA | Y | N | U | NA | Y | N | U | NA | Y | N | U | NA | Y | N | U | NA | Y | N | U | NA |  |
| Gebremedin, et al, 2009. | **√** |  |  |  | **√** |  |  |  | **√** |  |  |  | **√** |  |  |  | **√** |  |  |  | **√** |  |  |  | **√** |  |  |  |  | **√** |  |  |  | **√** |  |  |  | **√** |  |  | **√** |  |  |  | 8/11(72.7%) |

*Y=yes, N=no, U=unclear, NA=not applicable, <60%=low, 60-80%=medium, >80%=high quality.
